# Sgs1 and Exo1 Redundantly Inhibit Break-Induced Replication and *De Novo* Telomere Addition at Broken Chromosome Ends

**DOI:** 10.1371/journal.pgen.1000973

**Published:** 2010-05-27

**Authors:** John R. Lydeard, Zachary Lipkin-Moore, Suvi Jain, Vinay V. Eapen, James E. Haber

**Affiliations:** Department of Biology and Rosenstiel Basic Medical Sciences Research Center, Brandeis University, Waltham, Massachusetts, United States of America; The University of North Carolina at Chapel Hill, United States of America

## Abstract

In budding yeast, an HO endonuclease-inducible double-strand break (DSB) is efficiently repaired by several homologous recombination (HR) pathways. In contrast to gene conversion (GC), where both ends of the DSB can recombine with the same template, break-induced replication (BIR) occurs when only the centromere-proximal end of the DSB can locate homologous sequences. Whereas GC results in a small patch of new DNA synthesis, BIR leads to a nonreciprocal translocation. The requirements for completing BIR are significantly different from those of GC, but both processes require 5′ to 3′ resection of DSB ends to create single-stranded DNA that leads to formation of a Rad51 filament required to initiate HR. Resection proceeds by two pathways dependent on Exo1 or the BLM homolog, Sgs1. We report that Exo1 and Sgs1 each inhibit BIR but have little effect on GC, while overexpression of either protein severely inhibits BIR. In contrast, overexpression of Rad51 markedly increases the efficiency of BIR, again with little effect on GC. In *sgs1*Δ *exo1*Δ strains, where there is little 5′ to 3′ resection, the level of BIR is not different from either single mutant; surprisingly, there is a two-fold increase in cell viability after HO induction whereby 40% of all cells survive by formation of a new telomere within a few kb of the site of DNA cleavage. *De novo* telomere addition is rare in wild-type, *sgs1*Δ, or *exo1*Δ cells. In *sgs1*Δ *exo1*Δ, repair by GC is severely inhibited, but cell viaiblity remains high because of new telomere formation. These data suggest that the extensive 5′ to 3′ resection that occurs before the initiation of new DNA synthesis in BIR may prevent efficient maintenance of a Rad51 filament near the DSB end. The severe constraint on 5′ to 3′ resection, which also abrogates activation of the Mec1-dependent DNA damage checkpoint, permits an unprecedented level of new telomere addition.

## Author Summary

A chromosomal double-strand break (DSB) poses a severe threat to genome integrity, and budding yeast cells use several homologous recombination mechanisms to repair the break. In gene conversion (GC), both ends of the DSB share homology to an intact donor locus, and the break is repaired by copying the donor to create a small patch of new DNA synthesis. In break-induced replication (BIR), only one side of the DSB shares homology to a donor, and repair involves assembly of a recombination-dependent replication fork that copies sequences to the end of the template chromosome, yielding a nonreciprocal translocation. Both processes require that the DSB ends be resected by 5′ to 3′ exonucleases, involving several proteins or protein complexes, including Exo1 and Sgs1-Rmi1-Top3-Dna2. We report that ectopic BIR is inhibited independently by Sgs1 and Exo1 and that overexpression of Rad51 recombinase further improves BIR, while GC is largely unaffected. Surprisingly, when both Sgs1 and Exo1 are deleted, and resection is severely impaired, half of the cells acquire new telomeres rather than completing BIR or GC. New telomere addition appears to result from the lack of resection itself and from the fact that, without resection, the Mec1 (ATR) DNA damage checkpoint fails to inactivate the Pif1 helicase that discourages new telomere formation.

## Introduction

DNA double-strand breaks (DSBs) are generated by normal cellular processes including DNA replication or by exposure to DNA damaging agents or ionizing radiation. To maintain cell viability and preserve genomic integrity, cells employ multiple pathways of homologous recombination (HR) to repair DSBs [1]–[4]. A key initial step in HR is 5′ to 3′ resection of DSB ends to create single-stranded DNA (ssDNA) that recruits formation of a Rad51 filament, which engages in a search for homologous sequences. The predominant HR pathway is gene conversion (GC), a conservative mechanism in which both ends of the DSB share homologous sequences on a sister chromatid, a homologous chromosome, or at an ectopic location. Rad51-mediated strand invasion of the 3′-ended ssDNA allows the initiation of new DNA synthesis to copy a short region of the template and patch up the DSB. When only one DSB end shares homology to a template elsewhere in the genome, a less-efficient HR mechanism, break-induced replication (BIR), can be used to repair the break [5], [6]. In BIR, recombination is used to establish an uni-directional replication fork that can copy the template DNA to the end of the chromosome. If homologous sequences are located ectopically, BIR will result in formation of a non-reciprocal translocation with loss of the distal part of the broken chromosome and may be a significant source of gross chromosomal rearrangements (GCRs) and genomic instability [7]. BIR requires the non-essential subunit of the Polδ polymerase, Pol32, and all of the essential replication machinery except those excluisvely required for formation of the pre-replicative complex [8], [9]. BIR can be used to restart stalled or collapsed replication forks during DNA replication [10] and elongate telomeres in the absence of telomerase [8]. An alternative way to repair the DSB is through *de novo* telomere addition through the action of telomerase [11]–[13], although this is a very inefficient process that is improved by elimination of the Pif1 helicase [14].

Genetic and *in vivo* molecular biological experiments indicate that the early steps of GC and BIR are shared [15]–[17]. Following the generation of a DSB, the Tel1/ATM kinase is loaded at sites of DSBs in an Mre11-Rad50-Xrs2 (MRX)-dependent manner [18], [19]. Tel1 in turn phosphorylates MRX [20], [21]. The Sae2 and MRX proteins mediate the initial resection [22], [23] which is continued via two alternate pathways, one using the Exo1 nuclease and the other employing the multifunctional RecQ family helicase Sgs1, in concert with Top3, Rmi1 and the essential helicase/nuclease Dna2 [22]–[24]. DNA resection is also essential to activate the Mec1-dependent DNA damage checkpoint kinase cascade that triggers a cell cycle arrest, allowing time for the cell to repair the beak prior to mitosis [25].

Following resection, Rad51-mediated strand invasion of the donor template occurs with similar kinetics, but the initiation of DNA synthesis at the 3′-end of the invading strand is greatly delayed in BIR as compared to GC [16], [17]. Recently, Jain et al [16] showed that a “Recombination Execution Checkpoint” (REC) delays the initiation of BIR synthesis if a second DSB end has not become engaged nearby on the same template. It is unclear if the delay in BIR synthesis is due to a restructuring of the strand invasion D-loop and/or the recruitment of BIR associated proteins. The efficiency of BIR is inhibited by Sgs1, as there is an increase in BIR in *sgs1*Δ cells [16]. Sgs1 also has been shown to disrupt HR intermediates [26], inhibit homeologous recombination [27]–[29], and to dissolve double Holiday Junctions (dHJ) to yield noncrossovers [30]–[32].

To better understand the role of Sgs1 in BIR, we examined mutations of non-essential genes that either cooperate or act redundantly with Sgs1 in many of its roles in DNA metabolism, including DNA resection. Here we show that deletion of *SGS1* or *EXO1* increases the efficiency of BIR whereas overabundance of Sgs1 or Exo1 strongly inhibits it. Overexpression of Exo1 also inhibits GC. Deletion of other non-essential factors responsible for DNA resection, *TEL1* or *SAE2*, modestly increases the efficiency of BIR whereas deletion of MRX impairs BIR. Additionally, we find that overexpression of Rad51 markedly improves the efficiency of BIR but has little effect on GC. Finally, we show that Sgs1 and Exo1 redundantly prevent remarkably efficient *de novo* telomere addition at broken chromosome ends, a pathway dependent on both telomerase and Sae2.

## Results

### Assays to study break-induced replication and gene conversion in *S. cerevisiae*

**Figure 1:**
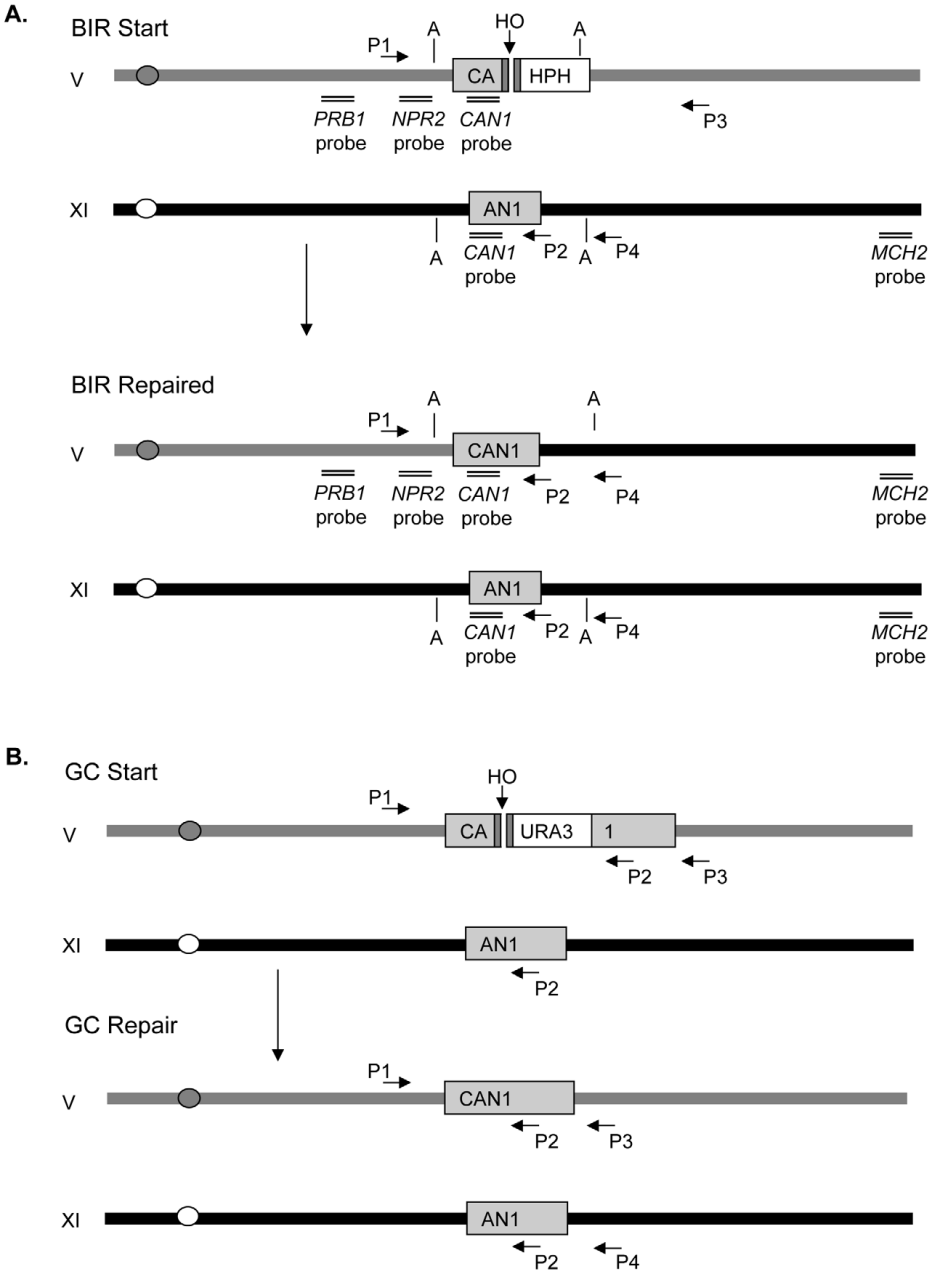
Experimental systems of break-induced replication (BIR) and gene conversion (GC). (A) In the experimental system to study BIR, an *HPHMX* marked HO cut site (gray bar) is integrated into the *CAN1* gene on Ch V, deleting the 3′ end portion of the gene, the remaining sequences are represented as CA. The AN1 donor sharing 1,157 bp homology with *CAN1* is integrated into Ch XI. PCR with primers P1 and P2 monitors the initiation of new DNA synthesis while PCR with primers P1 and P4 detects synthesis past the AN1 sequences, specifc to the donor sequences on Ch XI. Southern blot analysis of AvaI-digested (marked by “A”) DNA probed with *CAN1* sequences monitors extension of the BIR fork. Completion of BIR is monitored by Pulse-field gel electrophoresis (PFGE) followed by Southern blot analysis using the *MCH2* sequences that are duplicated when the entire donor chromosome arm is copied. (B) In the experimental system to study ectopic GC. A galactose inducible HO endonuclease generates a DSB within the *CAN1* locus (disrupted by *URA3* creating a 376 bp gap) on Ch V. An additional 2,404 bp of homologous sequences to the gene conversion donor sequences found on Ch XI are distal to the cut site and are denoted as “1.” PCR with primers P1 and P2 monitors both the starting strain and repair into the *CAN1* sequences. PCR with primers P1 and P3 monitors repair by GC in which the distal end of the break is retained.

To study BIR we used the haploid *Saccharomyces cerevisiae* strain JRL346. A galactose-inducible HO endonuclease is expressed to induce a DSB at a modified *CAN1* locus approximately 30 kb from the telomere in the non-essential terminal region on Chromosome V (Ch V) (Figure 1A). The HO endonuclease cut site and an adjacent hygromycin-resistant marker, *HPH-MX*, was integrated into the *CAN1* locus, deleting the 3′ portion of the gene but retaining the 5′ portion of the gene (denoted as CA). A 3′ portion of the gene (denoted as AN1) with 1,157 base pairs of shared homology to CA on Ch V was introduced in the same orientation into Ch XI, 30 kb from its telomere. Prior to HO induction, these cells are canavanine-resistant (Can^R^) because *CAN1* is disrupted. Completion of BIR results in a non-reciprocal translocation that duplicates the donor sequences and the more distal part of the left arm of Ch XI, thus restoring an intact *CAN1* gene. These cells become canavanine-sensitive (Can^S^) and hygromycin sensitive (Hph^S^). About 20% of cells are viable with 99.85% of these cells repairing by BIR and a small fraction by nonhomologous end-joining (NHEJ). The efficiency of BIR repair allows us to physically monitor the kinetics of repair by PCR, Southern blot and pulse-field gel electrophoresis (PFGE), as described in Materials and Methods.

**Figure 2:**
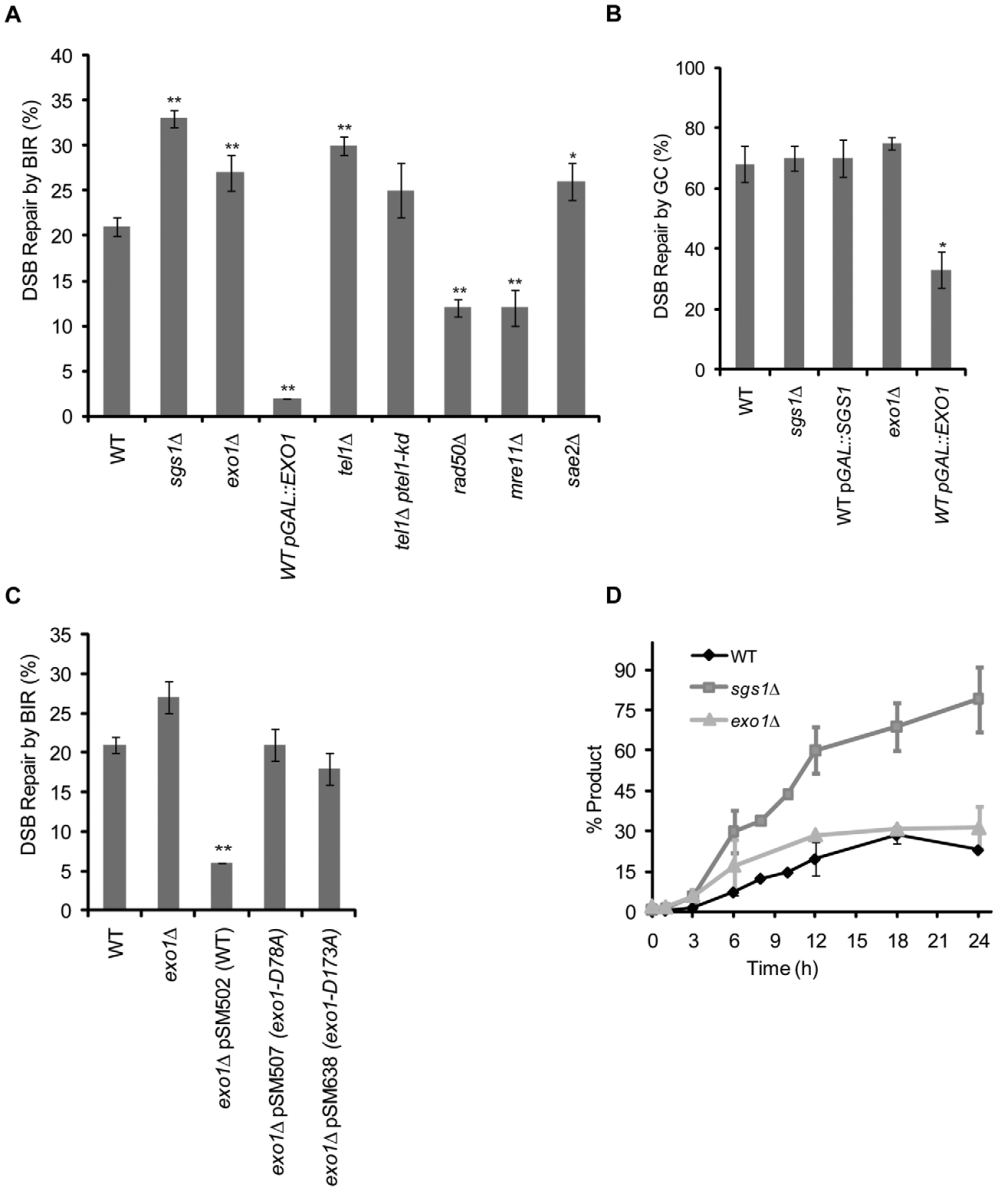
Sgs1 and Exo1 negatively regulate BIR. (A) Efficiency of BIR in cells as measured by viability following a DSB. (B) Efficiency of GC in cells as measured by viability following a DSB. (C) Efficiency of BIR in wild type (WT), *exo1*Δ, overexpression of *EXO1* and overexpression of *EXO1* nuclease-dead alleles measured by viability following a DSB. For (A–C), data are the mean ±standard error of the mean. Values marked with asterics are statistically significant (*represents p<0.05, ** represents p<0.01 compared to wild type). (D) The kinetics of repair are shown for PCR of BIR induced in cycling WT, *sgs1*Δ and *exo1*Δ cells amplified with P1 and P2 primer set labeled as “*CAN1*” and the standard *FLO9* locus of. Data are the mean ±standard deviation.

To compare the effects of mutations on GC, we used the isogenic strain JRL475 (Figure 1B). The GC strain was modified from the BIR strain by introducing 2,404 bp of homology marked by *URA3* to the other end of the break (denoted as 1, for the 3′-end of *CAN1*). The insertion of the *URA3*-1 sequences also deleted 376 bp in the middle of the *CAN1* so there is a gap between the homology shared by the two DSB ends created by HO cleavage (CA-*URA3*-1) with the donor sequences on Ch XI (AN1). Repair by GC results in restoration of the *CAN1* gene, rendering cells Can^S^, but, unlike BIR, the Ch V arm distal to the cut site is retained. When there is a second end of homology to a DSB break, the cell strongly favors GC over BIR [16], [17], [33], so that after induction of a DSB cell viability increases from 20% in the BIR strain to nearly 70% when there are two ends of homology and GC is used to repair the break (Figure 1B and Figure 2B).

### Deletion of *SGS1* increases the efficiency of BIR

To better understand the role of Sgs1 in BIR, we first measured the viability of *sgs1*Δ cells after inducing a DSB (Figure 1A). As previously shown [16], *sgs1*Δ cells are 1.5 times more efficient in BIR compared to wild type cells (Figure 2A), repairing the break with 33% efficiency (p<0.001). To confirm that the increase in viability directly correlates with an increase in repair product, we monitored the kinetics of repair using the PCR assay that detects the first 242 bp of new DNA synthesis. The maximum amount of product detected by PCR (18% at 12 hours) in wild type cells (Figure 2D) is comparative to the viability of cells (21%) following induction of the DSB (Figure 2A). As expected, deletion of *SGS1* increased the efficiency of product formation compared to wild type cells (Figure 2D). Using the previously described BIR system involving the *LEU2* sequences [16] we also showed that a helicase-dead allele of Sgs1 [34] behaves like the complete deletion of Sgs1 (Figure S1). We have previously shown that deletion of *sgs1*Δ does not increase the efficiency of GC events in which there is perfect homology or when there is a small gap in homology of 1.2 kb or less [16], [35]. We confirmed that *sgs1*Δ does not affect the efficiency of GC in the ectopic assay used here (Figure 1B and Figure 2B).

### The non-essential genes required for DNA resection affect the efficiency of BIR

To better understand the role of Sgs1 in BIR, we investigated a number genes that have previously been shown to interact genetically with Sgs1 [27], [29], [36]–[41]. Deletions of *MSH6*, *MUS81*, *YEN1*, *RAD27*, *ESC2*, *DIA2*, *YBR094w*, or *RNH202* did not have a statistically significant effect on BIR when tested for viability after inducing a DSB that can only be repaired by BIR (Table S1). However, we found that the other non-essential genes required for 5′ to 3′ resection of DSB ends all affect the efficiency BIR. A deletion of *SAE2* resulted in a slight, but statistically significant, increase in viability (p = 0.02). In contrast, deleting subunits of the MRX complex, *mre11*Δ or *rad50* Δ, decreased viability nearly 2 fold (both p = 0.003) (Figure 2A). The effect of deleting *mre11*Δ or *rad50*Δ is consistent with results previously seen in a diploid BIR assay in which a DSB is induced at the *MAT* locus on Ch III [17], [42], but differs from a transformation-based BIR assay that saw no requirement for MRX in BIR [15].

Because Tel1 plays a role in suppressing gross chromosomal rearrangements and enhances Sae2 and MRX activity in DNA resection [43] we asked if deletion of *TEL1* would affect BIR. Similar to *sae2*Δ, deletion of *TEL1* resulted in a small but statistically significant increase in viability (p = 0.008) (Figure 2A). Complementation of a *tel1*Δ strain with the kinase-dead allele [20] partially restored viability to wild type levels (Figure 2A).

The Exo1 nuclease acts redundantly with Sgs1 in DNA resection after the initial trimming of the ends by Sae2 and MRX, although by itself *exo1*Δ has a minimal impact on 5′ to 3′ resection [22]–[24]. Similar to *sgs1*Δ, deletion of *EXO1* (p = 0.001) increased viability nearly 1.5 times compared to wild type (Figure 2A). Also like *sgs1*Δ, deletion of *EXO1* increased the efficency of BIR when measured by PCR (Figure 2D) and does not affect the efficiency of GC (Figure 2B).

### Overexpression of both *SGS1* and *EXO1* inhibit BIR

**Figure 3:**
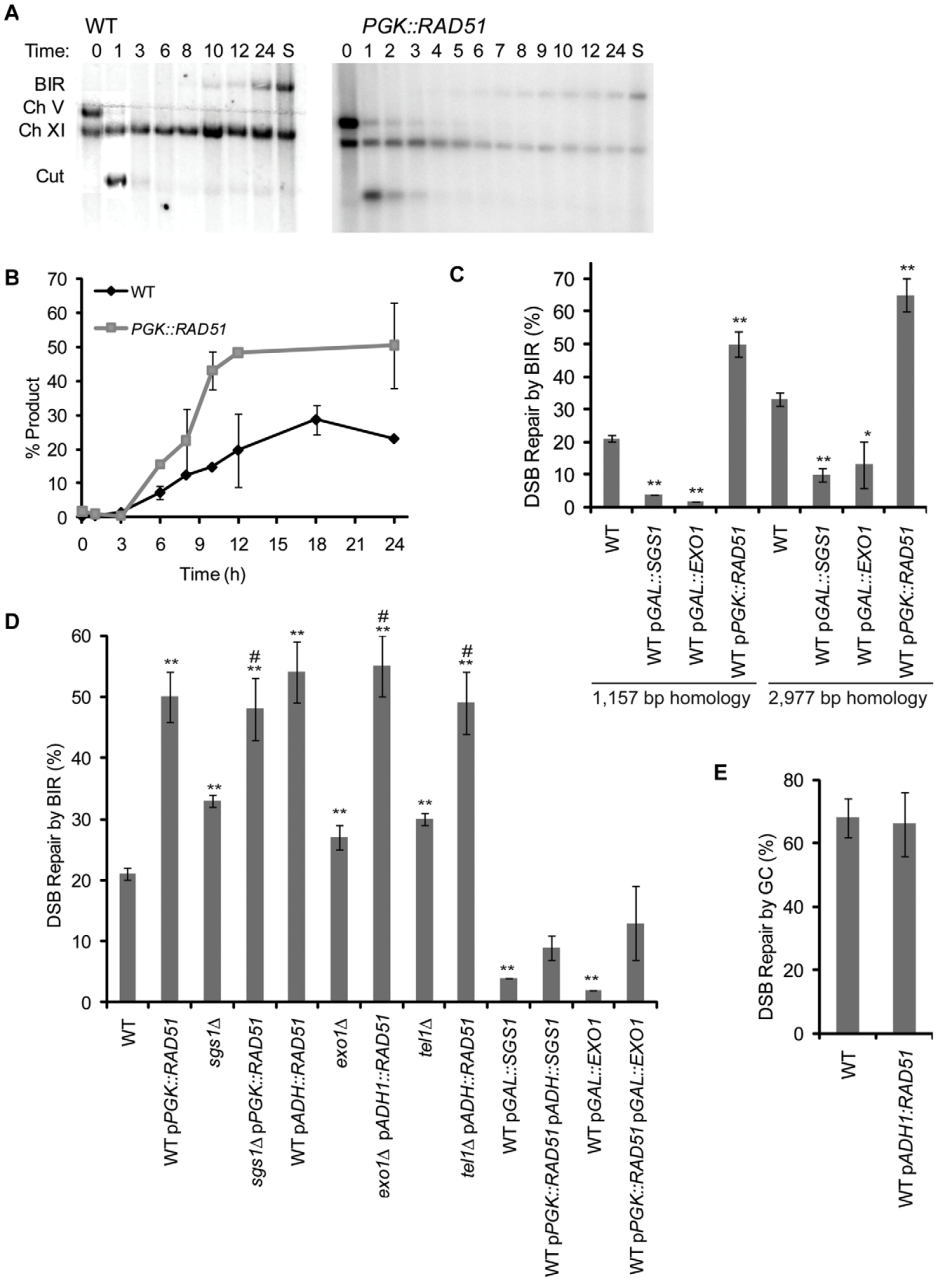
Overexpression of *RAD51* increases the kinetics and efficiency of BIR. (A) Southern blot analysis of the kinetics of repair product in wild type and p*PGK::RAD51* cycling cells as indicated in Figure 1A. Lane S contains DNA from a colony where BIR occurred. (B) Kinetics of repair are shown for PCR of BIR induced in cycling wild type (WT) and p*PGK::RAD51* cells. Data are the mean ±data range. (C) Efficiency of BIR in cells as measured by viability following a DSB in a BIR assay with increased homology (2,977 bp homology). Data from Figure 2A and Figure 3D (1,157 bp homology strain) are shown for comparison. Data are the mean ±s.e.m. Values marked with asterics are statistically significant (*represents p < 0.05, ** represents p < 0.01 compared to wild type). (D) Efficiency of BIR in strains graphed in Figure 2 also carrying either p*PGK::RAD51* or p*ADH::RAD51* as measured by viability following a DSB. Data are the mean ±s.e.m. Values marked with asterics or number sign are statistically significant (*represents p < 0.05, ** represents p < 0.01 compared to wild type. # represents p < 0.05 to the corresponding single mutant). (E) Efficiency of GC in WT and p*PGK::RAD51* as measured by viability following a DSB.

Plamids overexpressing Sgs1 pYES2-SGS1 [44] or Exo1 (pSL44) [45] were expressed under the control of a galactose-inducible promoter on a high copy plasmid. These overexpression plasmids are denoted as p*GAL::SGS1* and p*GAL::EXO1*, respectively. Expression is induced concomitantly with HO induction. In cells carrying p*GAL::SGS1*, the efficiency of BIR decreased 5 fold (p<0.001) whereas in p*GAL::EXO1* the efficiency of BIR decreased 10 fold (p<0.001) (Figure 2A). Overexpression of these genes did not affect cell viability in cells that lacked an HO cleavage site (data not shown). Furthermore, we found that Exo1 overexpression inhibited BIR prior to inhibition of new DNA synthesis, by monitoring the kinetics of repair by PCR (Figure S2). The strong inhibition of BIR by overexpressing Exo1 depends on the nuclease activity of this protein, as there is no such inhibition when we overexpressed plasmids carrying *exo1* mutations that are required for exonuclease activity (Figure 2C). As shown previously [8], increasing the homology in our BIR assay more than two fold to 2,977 bp increases the efficiency of BIR (Figure 3C). The increase in homology results in slightly higher viability but does not significantly suppress the effects of overexpressing *SGS1* or *EXO1* (Figure 3C). When tested in the GC assay, overexpressing Sgs1 had no effect on viability but overproduction of Exo1 decreased viability by half (Figure 2B).

### Overexpression of Rad51 increases the efficiency and kinetics of BIR

The initiation of BIR is delayed several hours after the ends of the DSB begin to be resected at a wild type rate of about 4 kb/hr [22], [46]. We have also previously shown that the abundance of Rad51 is sufficient to continuously coat only about 10 kb of ssDNA on either side of the break [47]; consequently it is possible that excess ssDNA would interfere with forming or maintaining a stable and efficient Rad51 filament that is needed to promote strand invasion and initiation of new DNA synthesis. Excess ssDNA has been previously shown to interfere with recombination in meiotic cells [48]. We therefore asked if overexpression of Rad51 would also increase the efficiency of BIR, using well-characterized high-copy plasmids in which *RAD51* was expressed under the *ADH1* promoter (pDBL(RAD51)) [49] or under the *PGK* promoter (pSJ5). Strikingly, overexpressing *RAD51* in wild type cells caused a 2.5-fold increase in viability (p<0.001) when expressed under control of either promoter (Figure 3D). When we tested the same plasmids in the GC assay we found that there was a slight but not statistically significant decrease in viability (Figure 3E). These results clearly indicate that Rad51 overexpression preferentially stimulates BIR. Overexpression of RAD51 in the BIR assay with longer homology further increased the efficiency of BIR (Figure 3C). We also find that the efficiency of BIR is increased when we tested the kinetics of repair by Southern blot (Figure 3A) and PCR (Figure 3B). However, when normalized to the percent of final product the kinetics of repair are not different from wild type cells (data not shown).

An elevated level of Rad51 increased the viability of *sgs1*Δ, *exo1*Δ or *tel1*Δ cells to the level seen for overexpressed *RAD51* alone (Figure 3D), so the effects of *RAD51* expression and deleting *SGS1* or *EXO1* are not additive. However, overexpressing *RAD51* in cells also overexpressing *SGS1* or *EXO1* did not significantly suppress the inhibition of BIR that is seen with overexpressing *SGS1* or *EXO1* alone (Figure 3D). These results could suggest that Sgs1 and Exo1 act prior to the rate-limiting step carried out by Rad51. In the case of Sgs1, it could be in dismantling transient strand invasion encounters; for Exo1, there is no evident mechanism at this point unless a modest increase in resection [45] would overwhelm excess Rad51.

### Sgs1 and Exo1 redundantly inhibit new telomere addition at DSBs

**Figure 4:**
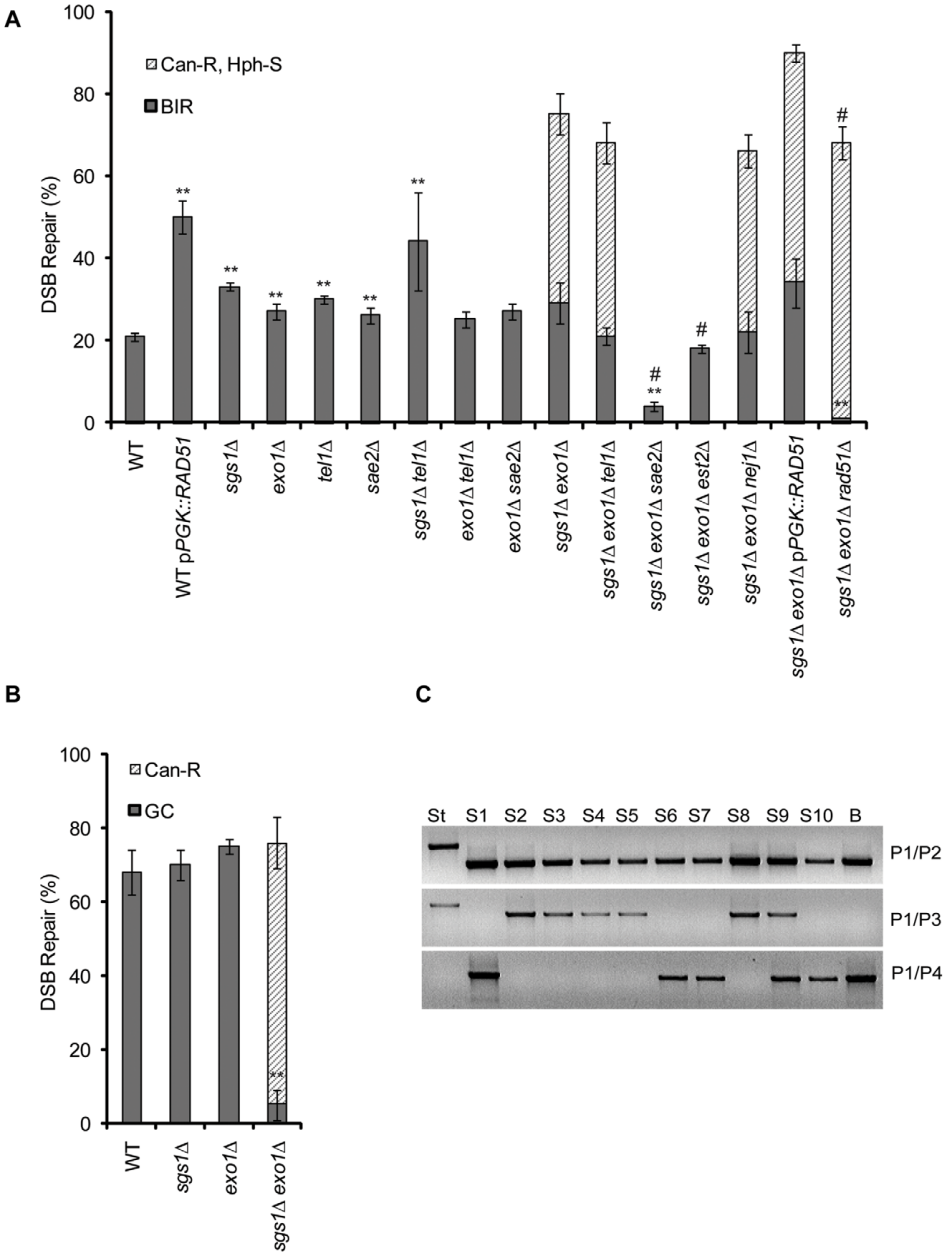
The effect of *sgs1*Δ *exo1*Δ on the viability and repair product in BIR and GC. (A) The viability and phenotypic characterization of wild type (WT), *sgs1*Δ, *exo1*Δ, *tel1*Δ, *sgs1*Δ*sgs1*Δ *exo1*Δ, p*PGK::RAD51* and indicated double and triple mutant combination cells following a DSB in the BIR assay. BIR colonies (Can^S^ Hph^S^) represent those that have repaired the DSB by BIR while Can^R^ Hph^S^ colonies represent those that have a truncated chromosome. Data are the mean ±s.e.m. Values marked with asterics or number sign are statistically significant (*represents p < 0.05, ** represents p < 0.01 compared to wild type BIR. # represents p < 0.05 to the *sgs1*Δ *exo1*Δ Can^R^ Hph^S^ colonies). (B) The viability and phenotypic characterization of cells following a DSB in the GC assay. HR colonies (Can^S^) represent those that have repaired by Homologus Recombination (either BIR or GC) while Can^R^ colonies represent those that have a truncated chromosome. Data are the mean ±s.e.m. ** represents p < 0.01 compared to wild type. (C) Repair of Can^S^ colonies in the GC assay as monitored by PCR. Included are the starting GC strain (ST), ten Can^S^ colonies (S1–S10) and a colony that has repaired by BIR (B). PCR with primers P1 and P2 detects the starting band and shift to smaller size upon repair into the *CAN1* sequences if repair occurs either by GC or BIR. PCR of primers P1 and P3 monitors retention of the distal end of the DSB and is indicative of repair by GC. PCR with primers P1 and P4 monitors repair specific to BIR (see Figure 1).

We examined a a dramatic 2-fold increase in viability in an *sgs1*D *exo1*D double mutant compared to *sgs1*Δ or *exo1*Δ alone when tested in the BIR assay (Figure 4A); however this increase is not in the level of BIR. Instead, it is due to a dramatic increase in new telomere addition, as described below. There is in fact no increase in BIR events compared to the single mutants and repair appears to be no better than wild type cells when repair was monitored by PCR (Figure S3). As has previoulsy been reported [22]–[24], we found that resection is severely impaired in *sgs1*Δ *exo1*Δ cells as evident by the persistence of the cut chromosome band seen by Southern blot (data not shown). Although *TEL1* and *SAE2* moderately inhibit BIR and are involved in DNA resection like *SGS1* and *EXO1* [50], deleting *TEL1* did not cause new telomere additions at the DSB when ablated in combination with *sgs1*Δ or *exo1*Δ nor did deletion of *SAE2* in combination with *exo1*Δ (Figure 4A).

### DSBS are frequently repaired by telomere addition in *sgs1Δ exo1Δ* cells

As mentioned above, when we analyzed the viablity of *sgs1*Δ *exo1*Δ cells, we found that half of the survivors did not have the Can^S^ Hph^S^ phenotype indicative of repair by BIR (Figure 4A). Instead, the new survivors were Hph^S^ but Can^R^, suggesting that they might have lost the terminal non-essential portion of Ch V distal to the cut site but failed to restore a functional *CAN1* locus.

**Figure 5:**
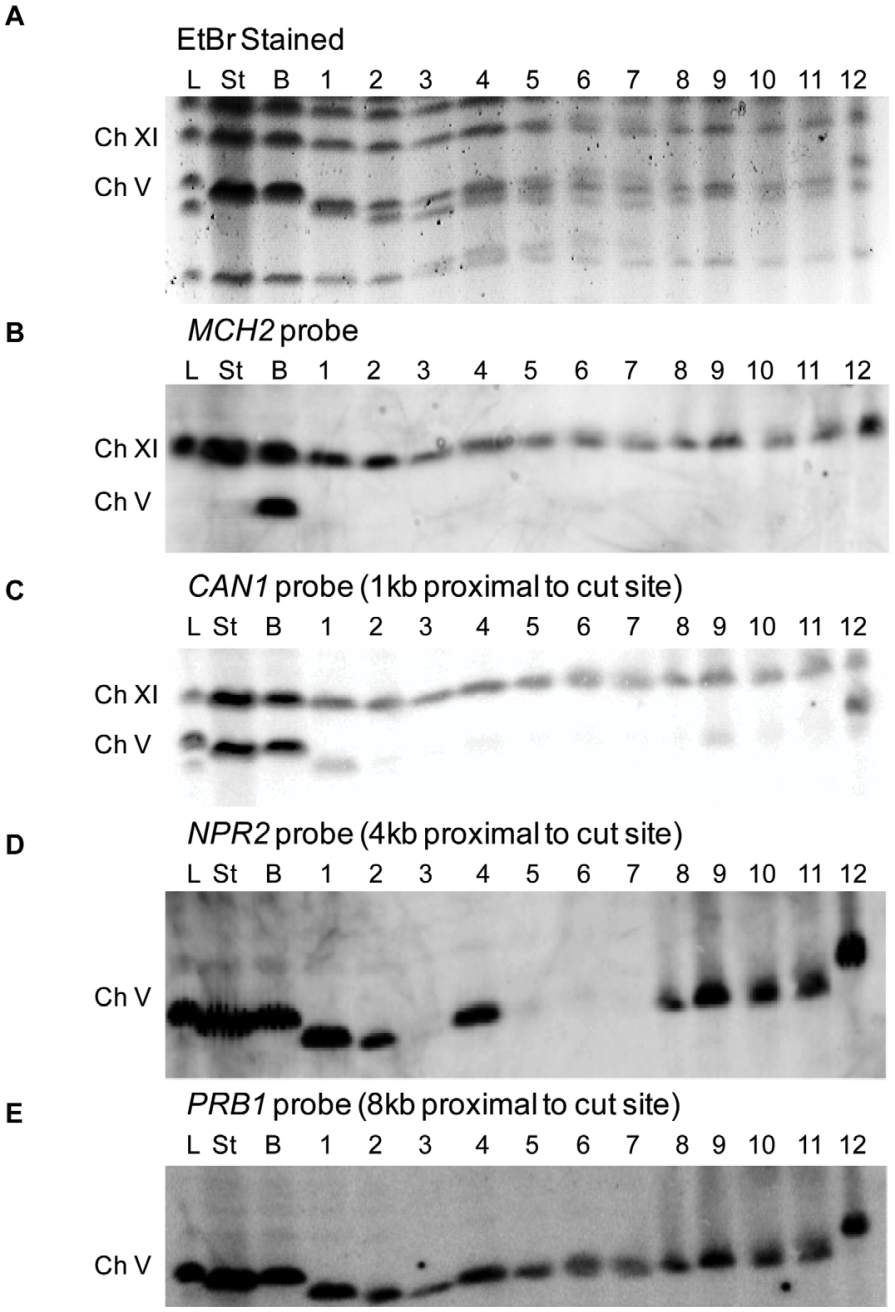
Characterization of Can^R^ HPH^S^ *sgs1*Δ *exo1*Δ colonies in the BIR strain by PFGE. (A) Ethidium bromide-stained agarose gel PFGE gel of *sgs1*Δ *exo1*Δ colonies that have repaired the DSB. Included are the ladder (L), starting strain prior to DSB induction (ST), Can^S^ HPH^S^ colony that has repaired by BIR (B), and twelve Can^R^ HPH^S^ colonies (1–12). Arrows indicate additional uncharacterized chromosomal fragments. (B) Southern blot analysis of (5A) by hybridization with a probe for *MCH2* that normally lies 6 kb from the telomere on Ch XI (See Figure 1). (C) The blot was stripped and Southern blot analysis was performed by hybridization with a probe for *CAN1* that normally lies 33 kb from the telomere on Ch V and is 1 kb proximal to the HO cut site (See Figure 1). (D) The blot was stripped and Southern blot analysis was performed by hybridization with a probe for *NPR2* that normally lies 36 kb from the telomere on Ch V and is 4 kb proximal to the HO cut site (See Figure 1). (E) Southern blot analysis was performed on (5D) by hybridization with a probe for *PRB1* that normally lies 40 kb from the telomere on Ch V and is 8 kb proximal to the HO cut site (See Figure 1).

Sgs1 has previously been shown to inhibit homeologous recombination [27], [29], specifically the formation of translocations between *CAN1* and two highly diverged CAN1 homologs, *LYP1* and *ALP1*, on Ch XIV [51]; these rearrangements might be further elevated by the absence of Exo1. Alternatively, given that *sgs1*Δ *exo1*Δ severely retards 5′ to 3′ resection, the chromosome end could be stabilized, allowing new telomere addition. To distinguish between these possibilities, we performed pulse field gel electrophoresis (PFGE) on 12 independent Can^R^ Hph^S^ colonies, comparing them to the starting strain and a survivor that repaired by BIR (Can^S^ Hph^S)^ (Figure 5). The ethidium bromide-stained agarose gel (Figure 5A) shows that the majority of the Can^R^ Hph^S^ survivors (lanes 1–11) have a smaller chromosome than the starting (ST) strain or one repaired by BIR (B). (There is no size difference in Ch V size prior to DSB induction and after BIR because the 30 kb of non-essential region distal to the cut site on Ch V is replaced by a duplication of 30 kb from Ch XI.) We confirmed by Southern blot that the band remaining at the original position of Ch V is Ch VIII, which is approximately the same size as Ch V in this strain background (data not shown). One Can^R^ Hph^S^ colony (lane 12) increased in size from the original strain. These data indicate the Can^R^ colonies are not due to mutations in a restored *CAN1* gene, and are therefore not repaired by BIR nor by NHEJ that could have deleted a small region including *HPH*. To confirm that none of the Can^R^ Hph^S^ colonies were repaired by BIR, we probed with the *MCH2* probe that hybridizes proximal to the telomere on Ch XI (Figure 5B). The *MCH2* probe hybridized to sequences on Ch XI in every sample, but only to Ch V in the Can^S^ Hph^S^ colony that repaired by BIR.

To determine what sequences of Ch V were retained in the Can^R^ Hph^S^ colonies, we next probed the blot with a *CAN1* probe that hybridizes to the donor sequences on Ch XI and just proximal (1 kb) to the cut site on Ch V (Figure 1A, Figure 5C). The *CAN1* probe hybridized to sequences on Ch XI in all samples and to Ch V in the starting and BIR strains, but only to three Can^R^ Hph^S^ colonies (1, 9 and 12). This result indicates that at least 1 kb of sequence was deleted in the 9 other Can^R^ Hph^S^ survivors. To determine approximately how much sequence was deleted in the other Can^R^ Hph^S^ colonies we probed the Southern blot with a *NPR2* probe that specifically hybridizes to Ch V 4 kb proximal to the cut site (Figure 1A and Figure 5D). In this case, the *NPR2* probe hybridized to all Can^S^ samples except lanes 3, 5, 6, and 7. When we probed with *PRB1* that hybridizes approximately 9 kb proximal to the cut site on Ch V, the probe hybridized to Ch V in all Can^S^ survivors (Figure 1A and Figure 5E). We also probed the blot with the highly diverged *ALP1* and *LYP1* sequences on Ch XIV with which *CAN1* forms translocations in *sgs1*Δ cells [51], but these sequences did not hybridize to the novel chromosome in lane 12 (data not shown). We have not explored further the structure of this translocation.

**Figure 6:**
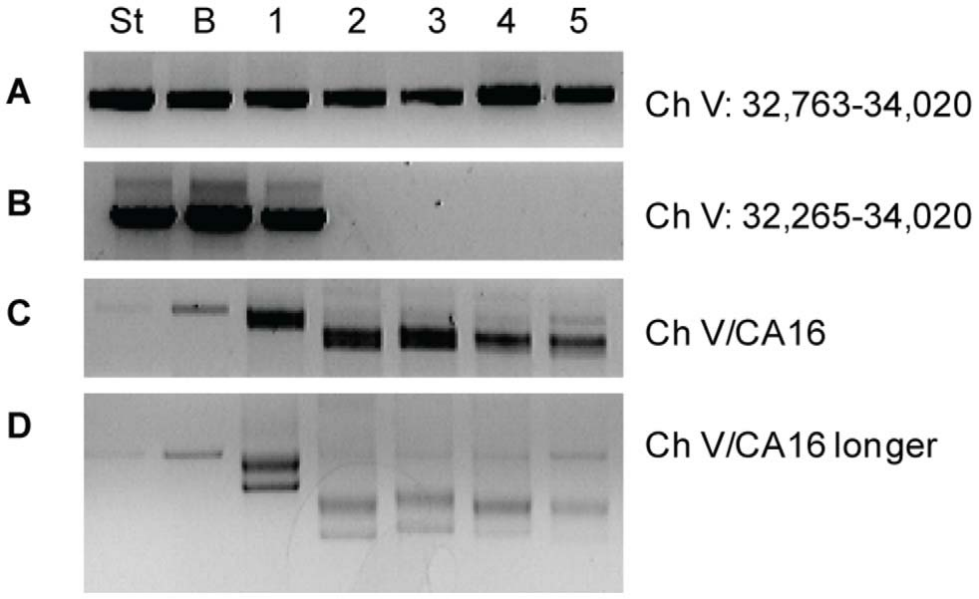
Marking of the breakpoint and detection of *de novo* telomere formation by PCR in *sgs1*Δ *exo1*Δ Can^R^ Hph^S^ cells. From the BIR assay. (A) PCR analysis of a starting strain prior to DSB induction (ST), Can^S^ Hph^S^ colony that has repaired by BIR (B), and five Can^R^ HPH^S^ colonies (1–5) with primers that amplify sequences (Ch V 32,763–34,020) approximately 750 bp proximal to the break. (B) PCR with primers that amplify sequences (Ch V 32,265–34,020) approximately 250 bp proximal to the break. (C) PCR with a Ch V-specific primer that amplifies all colonies indicated and primer CA16, a telomere-specific primer. (D) PCR product from 6C ran longer an agarose gel to better display the laddered PCR product indicative of *de novo* telomere formation in samples 1–5.

Based on our PFGE and Southern blot analysis we conclude that the great majority of the Can^R^ Hph^S^ survivors result in a truncation of Ch V after limited resection. To show if the sequences at the terminus of the truncations are indeed new telomeres, we determined the breakpoint of five independent *sgs1*Δ *exo1*Δ Can^R^ Hph^S^ repaired colonies by PCR, using a Ch V-specific primer and a telomere-specific primer as previously described [52], [53]. As shown in Figure 6, the presence of a new telomere is indicated by a laddered PCR product. We then sequenced the PCR product using the Ch V-specific primer. As shown in Table 1, all five *sgs1*Δ *exo1*Δ Can^R^ Hph^S^ colonies have new telomere sequences directly added to the Ch V sequences. Consistent with the PFGE and Southern blot analysis, the breakpoints were not at a uniform location. Based on our results, we hypothesize that in the absence of both Sgs1 and Exo1, a DSB frequently results in a truncated chromosome with newly added telomeres and that these additions can occur at several different sites, often as far as between 1 and 4 kb away from the DSB end. To confirm that these events are telomerase-dependent, we deleted *EST2*, an essential components of telomerase. As shown in Figure 4A, deletion of *EST2* does not affect repair by BIR but eliminates recovery of Can^R^ colonies.

**Table 1:** Sequenced breakpoints in *sgs1Δ exo1Δ* CAN^R^ HPH^S^ repaired colonies.

CAN^R^ Sample	Ch V Breakpoint	Sequence
1	32209	AAATTCCTGTCAAGGACCACCAAAG**GTGTGTGTGGTGTGTGGGTG**
2	32657	TTGGAGAAACCCAGGTGCCTGGGGT**GTGTGGGTG**
3	32636	TAAAAACGAAGGGAGGTTCTTAGGT**GTGTGGGTGTGGGTGT**
4	32657	TTGGAGAAACCCAGGTGCCTGGGGT**GTGGGTGTGGTGTG**
5	32708	GTTTTTGTATGGTTTGTGGTGCTGG**GTGTGGGTG**

The breakpoint in five independent *sgs1*Δ *exo1*Δ Can^R^ Hph^S^ repaired colonies were determined by PCR, amplified with Ch V-specific and telomere-specific primers (Figure 6), and sequenced as described [52], [53].

We next asked if NHEJ or HR pathways contributed to *de novo* telomere formation (Figure 4A). Telomere addition was not dependent on *NEJ1*, which is required for NHEJ. We next deleted *RAD51*, which is required for both BIR and GC. We confirmed that nearly all BIR is eliminated in *sgs1*Δ *exo1*Δ *rad51*Δ cells but also found a 20% increase in the number of cells with new telomeres. Although overexpression of *RAD51* increased the efficiency of BIR it did not suppress new telomere addition (Figure 4A). We then tested if the MRX-associated exonuclease Sae2 plays a role in new telomere addition. Recently, Sae2 and Sgs1 have also been shown to act in parallel telomere processing pathways [54]. Interestingly, when resection is nearly eliminated by deletion of *sae2Δ* in combination with *sgs1Δ exo1Δ*, new telomere addition is eliminated and BIR is significantly reduced (Figure 4A). When *TEL1* was deleted in combination with *sgs1Δ exo1Δ* there was no change in levels of BIR or *de novo* telomeres compared to *sgs1Δ exo1Δ* cells.

It has previously been seen that *sgs1*Δ *exo1*Δ cells are defective in GC when tested for the ability to successfully complete *MAT* switching [23]. When we tested the viability of *sgs1*Δ *exo1*Δ cells in our GC assay there was no discenrable effect on viability. However, when the phenotypes of the viable colonies were examined only 5% were Can^S^, which is indicative of repair by GC, while the remaining viabile colonies were Can^R^, consistent with a truncated chromosome (Figure 4B). The drastic decrease in GC is consistent with previously published defects seen in *sgs1*Δ *exo1*Δ cells. We analyzed 10 independent Can^S^ colonies by PCR to ascertain if the break was repaired by GC (Figure 4C). In fact, only 5 of the 10 colonies analyzed (samples S2, S3, S4, S5, S8) repaired by GC whereas 4 of the colonies repaired the break by BIR (S1, S6, S7, S10). One colony (S9) had PCR products consistent with repair by both GC and PCR. The use of BIR to repair half of the *sgs1Δ exo1Δ* colonies is consistent with the failure of these cells to activate the DNA damage checkpoint and thus to enter mitosis in the absence of DSB repair.

To verify that that the DNA damage checkpoint was impaired by the lack of normal 5′ to 3′ resection of the DSB ends we microscopically monitored the length of the cell cycle of individual cells plated on YEP-Gal to induce HO endonuclease, from the time that an unbudded G1 cell formed a bud until the dumbbell-shaped mother-daughter pair formed the next bud [55]. Wild type cells in which the DSB cannot be repaired remain arrested prior to anaphase for approximately 6 cell division times relative to an isogenic strain lacking the HO cleavage site [55]. In contrast, cells of the BIR strain lacking *SGS1*, *EXO1* and *RAD51*, so that they could not repair the DSB by homologous recombination, show a brief, but significant arrest. These cells extend the cell cycle 1.8 times the length of time of a derivative that lacks the cut site (6.2 h versus 3.5 h). Thus, there is still a brief activation of DSB-induced cell cycle arrest but much shorter than when extensive resection activates Mec1.

As was the case with Can^R^ *sgs1*Δ *exo1*Δ colonies found in the BIR assay, the Can^R^ colonies in the GC assay appear to be chromosome truncations with *de novo* telomere formation. PCR analysis showed that the broken chromosomes were truncated at different points proximal of the DSB (Figure S4). When representative isolates were tested by PCR as mentioned above we found that consistent with new telomere addition there was a laddered PCR product as seen in *sgs1*Δ *exo1*Δ cells in the BIR assay (Figure S4).

We conclude that eliminating both Sgs1 and Exo1, by markedly reducing 5′ to 3′ resection and most likely by preventing full activation of the Mec1-dependent DNA damage checkpoint (see Discussion), allows a dramatic increase in new telomere formation, rescuing almost half of all cells suffering a DSB.

## Discussion

In this work we show that the RecQ family helicase, Sgs1, and the Exo1 exonuclease negatively regulate BIR to maintain genomic integrity. From the observation that the efficiency of BIR was no greater in *sgs1*Δ *exo1*Δ than in a single mutant one might conclude that the helicase/endonuclease (Sgs1-Rmi1-Top3/Dna2) and Exo1 act in the same pathway, but since the *sgs1*Δ *exo1*Δ double mutant has such distinctly different phenotypes from *sgs1*Δ or *exo1*Δ it is difficult to know precisely why the double mutant does not show an increase in BIR similar to that seen when Rad51 is overexpressed in *sgs1*Δ or *exo1*Δ alone. We note also that other proteins responsible for 5′ to 3′ DNA resection, Sae2 and MRX, do not inhibit BIR in the same fashion; but the behavior of *sae2*Δ or *mre11*Δ may be explained by their other important roles in other steps in HR [1], [3],[4].

Sgs1 and Exo1 likely do not act in precisely the same way in inhibiting BIR. Sgs1-mediated inhibition of BIR may involve unwinding of a nascent strand invasion D-loop, as demonstrated *in vitro* for the human Sgs1 homolog, BLM [56], [57]. *In vivo* it is clear that the Sgs1 helicase can dismantle strand annealings and strand invasions if the heteroduplex DNA contains mismatches [27]–[29]. In meiotic recombination, Sgs1 prevents independent strand invasions of alternative templates [58], [59]. If Sgs1 dismantles heteroduplex DNA, we might expect that increased homology between the DSB end and the donor template would lead to a more stable D-loop that would counteract Sgs1. Increasing the extent of homology from 1.1 kb to ∼3 kb did not significantly change the response of cells to overexpression of Sgs1. It is also possible that Sgs1 inhibits the recruitment of some of the BIR-associated proteins. We note that the effect of deleting Sgs1 or Exo1 is not apparent in a different BIR assay system in a diploid in which nearly all homologous sequences distal to the DSB are deleted [17], [60]; and where there are 100 kb of homologous sequences centromere-proximal to the DSB that can be used to initiate BIR. However, even in this case, many BIR events fail to retain a marker 3 kb proximal to the DSB, suggesting either that more extensive homology increases BIR or that some more proximal sequences are especially favored in initiating BIR [61].

Rather than acting on D-loop stability, Exo1 may act on the assembly of the BIR replication fork. In response to DNA damage or defective checkpoint activation, Exo1 has also been shown to process stalled replication forks and resect nascent strands [62], [63]. The mechanism by which Exo1 interferes with fork integrity is unclear; it may be possible that the intermediate steps at which the BIR replication fork is assembled are an Exo1 substrate. We have previously shown that overexpression of Exo1 increases the rate of resection [45]; this has not been tested for Sgs1 overexpression.

A unifying hypothesis would be that BIR is severely limited if resection of the DSB ends is too extensive. There is a limited amount of Rad51 in the cell (about 3,500 molecules), enough to cover continuously about 10 kb of ssDNA [47]. Although Rad51 will initially form a filament with sequences close to the DSB (including the relevant “CA” sequences that engage in BIR), as resection proceeds the continuous polymerization and depolymerization of Rad51 may leave patches of Rad51 along much of the ssDNA so that by the time BIR is seen, many DSBs will not have a continuous Rad51 filament near the 3′ end to promote the completion of recombination. Thus, even in wild type cells, overexpressing Rad51 would ensure that there would be a functional filament over the CA sequences and BIR would consequently be more efficient. Deletions of Sgs1 or Exo1 would partially suppress the problem by slowing down resection (hence BIR is increased 1.5 times wild type), although we again note that *exo1*Δ by itself has little visible effect on resection. Overexpression of Rad51 is apparently unable to suppress the consequences of overexpressing Exo1 or Sgs1. It is important to note that Exo1 overexpression is only effective if nuclease activity is preserved; at least some of Exo1's functions in meiosis are independent of nuclease activity (N. Hunter, personal communication; L. Symington, personal communication). Increasing homology in our assay does not suppress these effects but further increases in homology may do so, as noted above.

It is possible that overexpressing Rad51 could ensure that the 3′-ended single-stranded DNA was better protected against degradation over the long time required to enact BIR, as previously suggested [64]. However, we have previously shown that in single-strand annealing where one of the flanking 1-kb homologies is very close to the DSB and the other is exposed only after 6 hr of 5′ to 3′ resection, at least 85% of cells are able to accomplish SSA, which would be impossible if even 1 kb of the 3′-end were degraded in the 6-hr period. Moreover, SSA was equally possible with and without Rad51 [16], arguing that Rad51 did not provide end-protection to the 3′-ended single-strand.

Eliminating both Sgs1 and Exo1 had a marked defect in completing GC but did not impair BIR so severely. Because resection is severely impaired in the *sgs1*Δ *exo1*Δ double mutant, it is possible that the more severe defect in GC is attributable to the need to resect more than 1 kb of intervening sequence before the “1” end of homology would be single-stranded (see Figure 1B). However, it is also possible that the difference reflects still another defect in *sgs1*Δ *exo1*Δ strains, a failure to activate the DNA damage checkpoint because of a lack of sufficient ssDNA [25], [65]. If mitosis is not arrested, then cells that have an unrepaired DSB will proceed through mitosis. This may lead to the loss of the acentric fragment, as we have shown in other assays [66], so that only the centromere-proximal DSB end will be inherited. This situation is not fatal for BIR, which only uses homology on that side of the DSB; indeed previous studies [17], [67] have shown that BIR may actually increase in a checkpoint-deficient situation whereas GC will be defective. Thus, even when GC should be possible, half of the HR outcomes of the *sgs1Δ exo1Δ* GC assay proved to be BIR events.

Strikingly, Sgs1and Exo1 also redundantly inhibit new telomere formation. In a previous study [12], when an HO-induced DSB was generated in a *rad52*Δ strain that could not carry out recombination but had apparently normal 5′ to 3′ resection, only about 1% of cells created new telomeres, and this was only in a situation where a “seed” of T_2_G_4_ telomere sequences was located centromere-proximal to the DSB. In the absence of the T_2_G_4_ repeats, new telomeres arose less than 0.1% of the time. The remarkably high level of new telomere formation (up to 50% of all cells) must be attributable to the elimination of vigorous resection in the double mutant strain, but it is also likely that the failure to activate the Mec1 DNA damage checkpoint also plays a key role. Recently, Makovets and Blackburn [68] have shown that the Pif1 helicase, which antagonizes new telomere formation [69], is phosphorylated in a Mec1-dependent fashion; hence if *sgs1*Δ *exo1*Δ block resection and that prevents Mec1 activation, new telomeres should increase. However, in the assay used by Makovets and Blackburn [68] the level of new telomeres added near an HO endonuclease-induced DSB was only about 2%. Moreover, Chung et al [60] also find that new telomere addition is much less efficient in cells lacking *MEC1* compared to *sgs1*Δ *exo1*Δ cells. Hence, it is likely that the 40–50% level of *de novo* telomere formation we find reflects both the failure to activate Pif1 when the checkpoint is not strongly activated and the severe block on resection itself.

Apparently *de novo* telomere formation does not require the recruitment of the MRX-Tel1 complex, as a *tel1*Δ mutant does not affect the formation of new telomeres in an *sgs1*Δ *exo1*Δ strain. When resection is blocked by deletion of *SAE2* in *sgs1*Δ *exo1*Δ cells, new telomeres are absent. The fact that new telomeres were added as far as 4 kb from the DSB site indicates that there is a residual resection activity that–over a period of perhaps many hours–can chew away the chromosome end and expose sites suitable for new telomere addition. However, we show that the MRX-asociated endonuclease SAE2 is required for *de novo* telomere formation.

In this work we have expanded our understanding of the genetic relationships of factors that negatively regulate BIR. Furthermore, we have provided evidence for a novel repair pathway that is redundantly impaired by Sgs1 and Exo1. Understanding the interplay of these factors in response to DNA damage and uncovering the molecular details of signaling between them to maintain genomic integrity will be an area of much future research.

## Materials and Methods

### Strains and plasmids

The wild type JRL346 was derived from JRL092 [8] by first disrupting the *LEU2* marker with a *leu2::hisG* construct from pNKY85 [70] to generate strain JRL187. The *HMRa-stk* gene was then knocked out with an *hmr::ADE3* fragment generated by PCR with mixed oligos to generate JRL346. All strains used to study BIR are isogenic to JRL346 and were created by standard gene disruption methods and confirmed by PCR unless otherwise stated [71]. In order to generate an assay to study GC that is isogenic with JRL346, an HOcs-*HPH* cassette [8] was integrated into Ch V between nucleotides 31,644 and 32,020, resulting in a truncation of the *CAN1* ORF at nucleotide 1,146 to create strain JRL017 (CL11-7 *can1,1-1446::HOcs::HPH)*. JRL017 was then modified by transforming in a *hphmx::URA3* “marker swap” cassette [72] to generate JRL472 (CL11-7 *can1,1-1446::HOcs::URA3::AVT2*). To introduce another 2,404 bp of homology to the donor, the *can1,1-1446::HOcs::URA3::AVT2* region with Ch V sequences 29,146 to 32,976 was amplified from JRL472 and integrated distal to the HO cut site into Ch V in strain JRL346 to generate JRL475 (*can1,1-1446::HOcs::URA3::AVT2 ykl215c::leu2::hisG::can1DEL1-289::AVT2*). As a result, there are Ch V sequences 33,177–32,020 shared between the donor and sequences proximal to the break, Ch V sequences 31,644–29,240 shared between the donor and sequences distal to the break and a 376 bp gap of homology. All mutant strains were created by standard gene disruption methods and confirmed by PCR. Plasmid pSJ5 was constructed by subcloning a XhoI-NotI fragment containing the *RAD51* ORF under the PGK promoter form pNSU256 [47] into pRS314 [73].

The wild type JRL346 was derived from JRL092 [8] by first disrupting the *LEU2* marker with a *leu2::hisG* construct from pNKY85 [70] to generate strain JRL187. The *HMRa-stk* gene was then knocked out with an *hmr::ADE3* fragment generated by PCR with mixed oligos to generate JRL346. All strains used to study BIR are isogenic to JRL346 and were created by standard gene disruption methods and confirmed by PCR unless otherwise stated [71]. In order to generate an assay to study GC that is isogenic with JRL346, an HOcs-*HPH* cassette [8] was integrated into Ch V between nucleotides 31,644 and 32,020, resulting in a truncation of the *CAN1* ORF at nucleotide 1,146 to create strain JRL017 (CL11-7 *can1,1-1446::HOcs::HPH)*. JRL017 was then modified by transforming in a *hphmx::URA3* “marker swap” cassette [72] to generate JRL472 (CL11-7 *can1,1-1446::HOcs::URA3::AVT2*). To introduce another 2,404 bp of homology to the donor, the *can1,1-1446::HOcs::URA3::AVT2* region with Ch V sequences 29,146 to 32,976 was amplified from JRL472 and integrated distal to the HO cut site into Ch V in strain JRL346 to generate JRL475 (*can1,1-1446::HOcs::URA3::AVT2 ykl215c::leu2::hisG::can1DEL1-289::AVT2*). As a result, there are Ch V sequences 33,177–32,020 shared between the donor and sequences proximal to the break, Ch V sequences 31,644–29,240 shared between the donor and sequences distal to the break and a 376 bp gap of homology. All mutant strains were created by standard gene disruption methods and confirmed by PCR. Plasmid pSJ5 was constructed by subcloning a XhoI-NotI fragment containing the *RAD51* ORF under the PGK promoter form pNSU256 [47] into pRS314 [73].

### Viability measurements

Logarithmically growing cells grown in YEP+2% Raffinose, or the appropriate drop-out media +2% Raffinose, were plated on either YEPD or YEP-Gal, and grown into colonies. Colonies were counted and were then replica plated onto plates containing either canavanine or hygromycin to confirm repair occurred by BIR. Experiments were performed at least 5 times for each strain unless otherwise indicated. To determine the statistical significance between strains the student's t-test was used (paired, two-tailed, n≥4 for all strains).

### HO induction and measurement of kinetics of DSB repair

Strains were grown in YEP+2% Raffinose to a cell density of 3×10e6 to 1×10e7 cells/mL. A 50 mL aliquot of cells was removed for the zero time point. Freshly made galactose was added to final concentration of 2% to induce HO expression. Cell aliquots were taken at the indicated time points throughout the time course.

### DNA analysis

PCR analysis of BIR was performed as previously described [8]. Briefly, we monitor the initiation of new BIR DNA synthesis using a PCR assay in which one primer is specific to Ch V and the other primer is specific to the donor sequence on Ch XI. Once a covalent molecule is formed, corresponding to the first 242 bp of new DNA synthesis, we see PCR product. At least three PCR reactions from three different experiments were performed for wild type, *sgs1*Δ and *exo1*Δ strains. For all other strains tested, at least three PCR reactions from two experiments were performed. The technical replicates from each biological experiment was first averaged and then the technical averages were averaged among the two experiments to obtain a biological average. Data were graphed as the biological averages normalized to the maximum product obtained by amplifying DNA from a strain that has repaired the DSB by BIR. Error bars represent the data range between the biological averages.

Repair is also measured by Southern blot that detects approximately the first 3 kb of new DNA synthesis was performed as previously described [8]. The analysis by Southern blot or pulse-field (CHEF) gel electrophoresis followed by Southern blot was performed as described [8] using the probes indicated in Figure 1. The breakpoints and sequences of *sgs1*Δ *exo1*Δ Can^R^ Hph^S^ repaired colonies were performed as described [52], [53].

## Supporting Information

Figure S1 The helicase-domain of Sgs1 is required to inhibit BIR. (A) In this assay to study BIR, an HO cut site is integrated into an ectopically located *LEU2* gene on Chromosome V (Ch V) in which the 3′ end portion of the gene is deleted, the remaining sequences are represented as LE. The donor sequences are the endogenous *LEU2* gene on Ch III. Repair of the DSB only occurs by BIR resulting in duplication of the *LEU2* gene and the distal sequences on Ch III. (B) Efficiency of BIR as measured by viability following a DSB in wild type (WT), *sgs1*Δ, or *sgs1*Δ cells complemented with a plasmid expressing the sgs1-hd allele (p*sgs1-hd*).
(0.21 MB TIF)

Figure S2 Overexpression of *EXO1* inhibits BIR. Kinetics of repair are shown for PCR assays of BIR induced in cycling wild type (WT) a*nd GAL::EXO1* cells. Data are the mean ±data range.
(0.13 MB TIF)

Figure S3 The efficiency of BIR is not increased in *sgs1Δ exo1Δ* cells. Kinetics of repair are shown for PCR assays of BIR induced in cycling wild type (WT) and *sgs1Δ exo1Δ* cells. Data are the mean ± data range for two experiments.
(0.16 MB TIF)

Figure S4 Marking of the breakpoint and detection *of de novo* telomere formation by PCR in *sgs1*Δ *exo1*Δ CAN^R^ survivors from the GC assay. (A) PCR analysis of a starting strain prior to DSB induction (ST), Can^S^ colony that has repaired by HR (S), and ten Can^R^ colonies (R1–R10) with primers that amplify sequences (Ch V 39,744–42,157) approximately 7.7 kb proximal to the break. (B) PCR with primers that amplify sequences (Ch V 34,271–37,985) approximately 2.2 kb proximal to the break. (C) PCR with primers that amplify sequences (Ch V 33,007–35,272) approximately 1 kb proximal to the break. (D) PCR with primers that amplify sequences (Ch V 32,265–34,020) approximately 250 bp proximal to the break. (E) PCR with a Ch V-specific primer that amplifies all colonies indicated and primer CA16, a telomere-specific primer.
(1.41 MB TIF)

Table S1 The effect of varied mutants on the efficiency of BIR. The viability of cells that could repair a DSB by BIR as shown in Figure 1A was compared by plating cells on YEP-galactose to induce expression of HO endonuclease and on YEPD, as described in Materials and Methods.
(0.07 MB DOCX)
