# Supplementary figures and images for: Sgs1 and Exo1 Redundantly Inhibit Break-Induced Replication and De Novo Telomere Addition at Broken Chromosome Ends
Source: PLoS Genet. 2010 May 27;6(5):e1000973. doi: 10.1371/journal.pgen.1000973 (PMC2877739; doi:10.1371/journal.pgen.1000973)

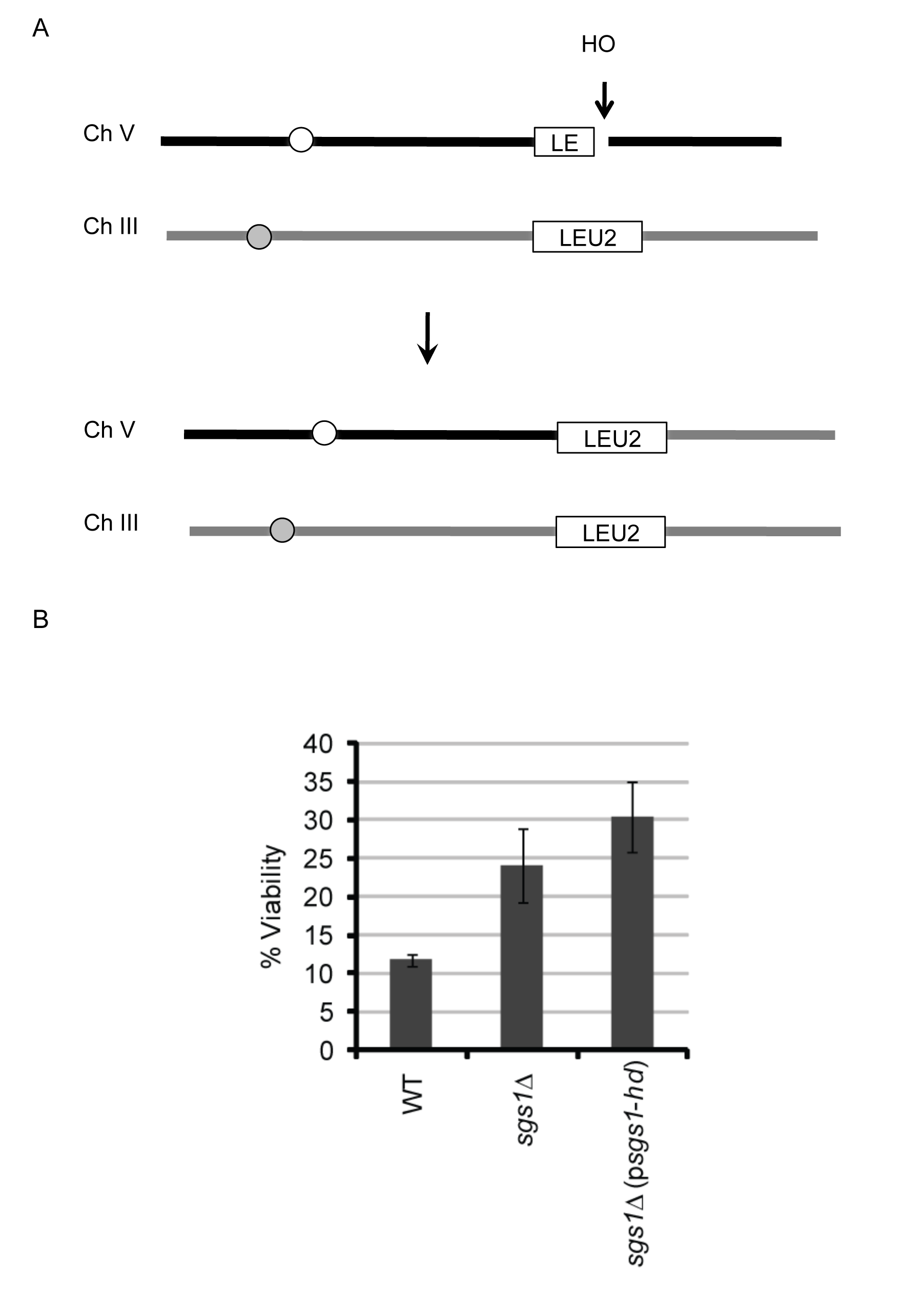

Supplement: Figure S1 — The helicase-domain of Sgs1 is required to inhibit BIR. (A) In this assay to study BIR, an HO cut site is integrated into an ectopically located LEU2 gene on Chromosome V (Ch V) in which the 3′ end portion of the gene is deleted, the remaining sequences are represented as LE. The donor sequences are the endogenous LEU2 gene on Ch III. Repair of the DSB only occurs by BIR resulting in duplication of the LEU2 gene and the distal sequences on Ch III. (B) Efficiency of BIR as measured by viability following a DSB in wild type (WT), sgs1Δ, or sgs1Δ cells complemented with a plasmid expressing the sgs1-hd allele (psgs1-hd). (0.21 MB TIF) [file pgen.1000973.s001.tif]

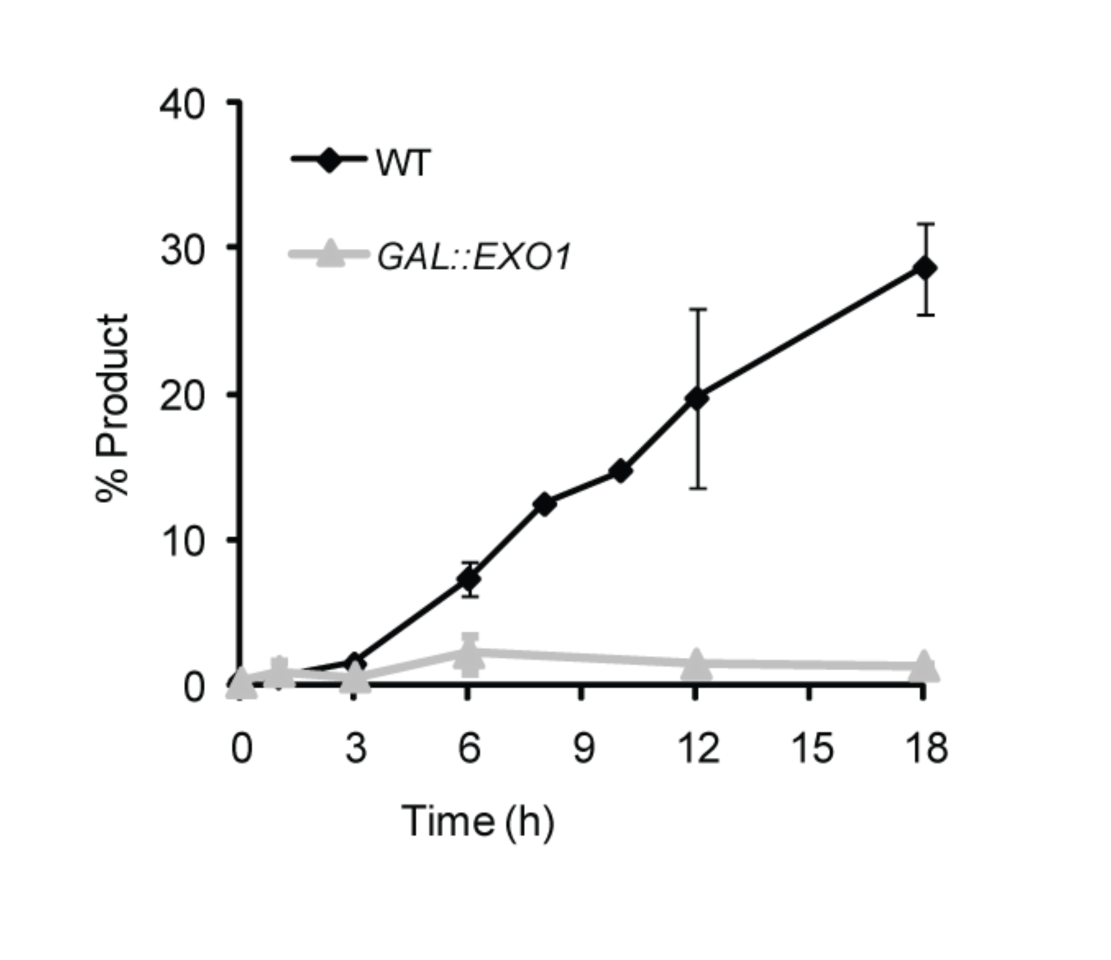

Supplement: Figure S2 — Overexpression of EXO1 inhibits BIR. Kinetics of repair are shown for PCR assays of BIR induced in cycling wild type (WT) and GAL::EXO1 cells. Data are the mean ±data range. (0.13 MB TIF) [file pgen.1000973.s002.tif]

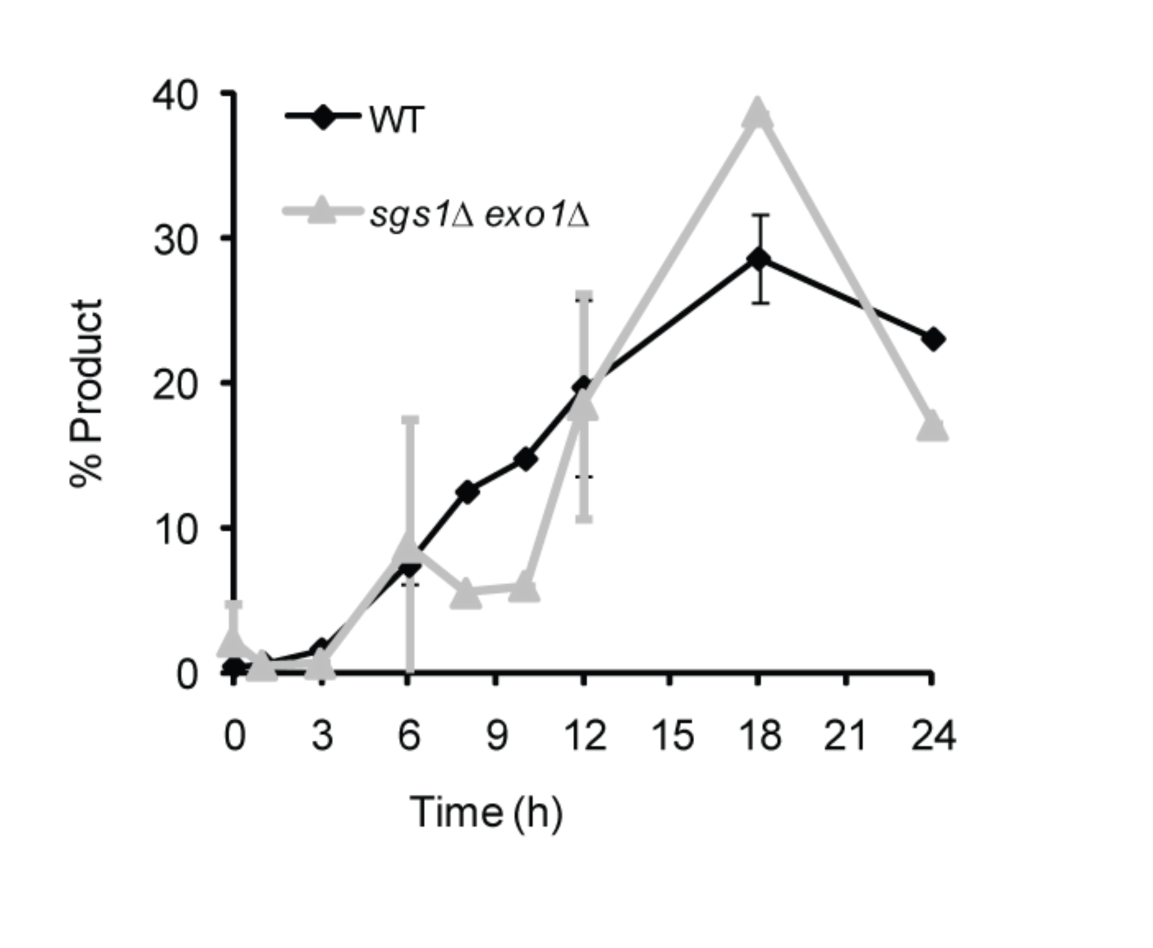

Supplement: Figure S3 — The efficiency of BIR is not increased in sgs1Δ exo1Δ cells. Kinetics of repair are shown for PCR assays of BIR induced in cycling wild type (WT) and sgs1Δ exo1Δ cells. Data are the mean ± data range for two experiments. (0.16 MB TIF) [file pgen.1000973.s003.tif]

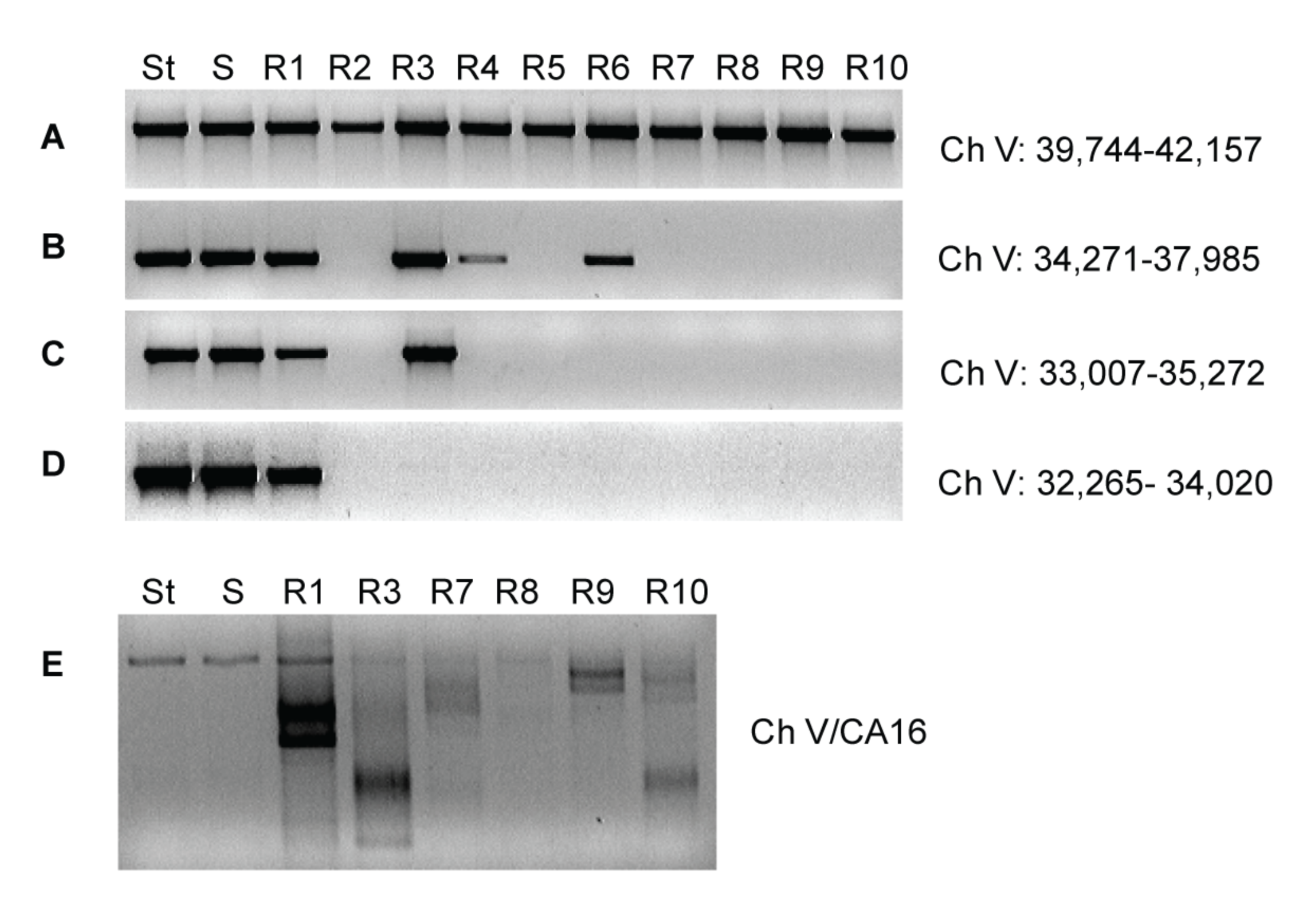

Supplement: Figure S4 — Marking of the breakpoint and detection of de novo telomere formation by PCR in sgs1Δ exo1Δ CANR survivors from the GC assay. (A) PCR analysis of a starting strain prior to DSB induction (ST), CanS colony that has repaired by HR (S), and ten CanR colonies (R1–R10) with primers that amplify sequences (Ch V 39,744–42,157) approximately 7.7 kb proximal to the break. (B) PCR with primers that amplify sequences (Ch V 34,271–37,985) approximately 2.2 kb proximal to the break. (C) PCR with primers that amplify sequences (Ch V 33,007–35,272) approximately 1 kb proximal to the break. (D) PCR with primers that amplify sequences (Ch V 32,265–34,020) approximately 250 bp proximal to the break. (E) PCR with a Ch V-specific primer that amplifies all colonies indicated and primer CA16, a telomere-specific primer. (1.41 MB TIF) [file pgen.1000973.s004.tif]
