# Supplementary material for: Sgs1 and Exo1 Redundantly Inhibit Break-Induced Replication and De Novo Telomere Addition at Broken Chromosome Ends
Source: PLoS Genet. 2010 May 27;6(5):e1000973. doi: 10.1371/journal.pgen.1000973 (PMC2877739; doi:10.1371/journal.pgen.1000973)
Supplement: Table S1 — The effect of varied mutants on the efficiency of BIR. The viability of cells that could repair a DSB by BIR as shown in Figure 1A was compared by plating cells on YEP-galactose to induce expression of HO endonuclease and on YEPD, as described in Materials and Methods. (0.07 MB DOCX) [file pgen.1000973.s005.docx]

**Table S1. The effect of varied mutants on the efficiency of BIR**

| **Strain** | **Relevant Genotype** | **Percent BIR** |
| --- | --- | --- |
| JRL346 | Wild type | 21±4 |
| JRL214 | *rad27Δ* | 21±1 |
| JRL217 | *msh6Δ* | 25±2 |
| JRL266 | *ybr094wΔ* | 20±1 |
| JRL349 | *rnh202Δ* | 31±4 |
| JRL354 | *esc2Δ* | 20±1 |
| JRL370 | *mus81Δ* | 24±3 |
| JRL385 | *yen1Δ* | 21±1 |
| JRL386 | *mus81Δ yen1Δ* | 22±3 |
| JRL532 | *dia2Δ* | 20±1 |

The viability of cells that could repair a DSB by BIR as shown in Figure 1A was compared by plating cells on YEP-galactose to induce expression of HO endonuclease and on YEPD, as described in Material and Methods.
